# Inactivation of avian influenza viruses by hydrostatic pressure as a potential vaccine development approach

**DOI:** 10.1099/acmi.0.000220

**Published:** 2021-04-13

**Authors:** Shana Priscila Coutinho Barroso, Ana Clara Vicente dos Santos, Patrícia Souza dos Santos, José Nelson dos Santos Silva Couceiro, Davis Fernandes Ferreira, Dirlei Nico, Alexandre Morrot, Jerson Lima Silva, Andrea Cheble de Oliveira

**Affiliations:** ^1^​ Laboratório de Termodinâmica de Proteínas e Estruturas Virais Gregorio Weber, Programa de Biologia Estrutural, Instituto de Bioquímica Médica Leopoldo de Meis, Universidade Federal do Rio de Janeiro, 21941-590, Rio de Janeiro, RJ, Brazil; ^2^​ Instituto Nacional de Ciência e Tecnologia de Biologia Estrutural e Bioimagem, Brazil; ^3^​ Laboratório de Biologia Molecular, Instituto de Pesquisas Biomédicas, Hospital Naval Marcílio Dias, Marinha do Brasil, Brazil; ^4^​ Centro Universitário IBMR, Rio de Janeiro, RJ, Brazil; ^5^​ Departamento de Virologia, Instituto de Microbiologia Professor Paulo de Góes, Universidade Federal do Rio de Janeiro, Rio de Janeiro, RJ, Brazil; ^6^​ Laboratório de Imunoparasitologia, Instituto Oswaldo Cruz, Rio de Janeiro, RJ, Brazil; ^7^​ Faculdade de Medicina, Departamento de Clínica Médica, Centro de Pesquisa em Tuberculose,, Universidade Federal do Rio de Janeiro, Rio de Janeiro, RJ, Brazil

**Keywords:** influenza virus, stability, high pressure, fluorescence spectroscopy, viral inactivation

## Abstract

Vaccines are a recommended strategy for controlling influenza A infections in humans and animals. Here, we describe the effects of hydrostatic pressure on the structure, morphology and functional characteristics of avian influenza A H3N8 virus. The effect of hydrostatic pressure for 3 h on H3N8 virus revealed that the particles were resistant to this condition, and the virus displayed only a discrete conformational change. We found that pressure of 3 kbar applied for 6 h was able to inhibit haemagglutination and infectivity while virus replication was no longer observed, suggesting that full virus inactivation occurred at this point. However, the neuraminidase activity was not affected at this approach suggesting the maintenance of neutralizing antibody epitopes in this key antigen. Our data bring important information for the area of structural virology of enveloped particles and support the idea of applying pressure-induced inactivation as a tool for vaccine production.

## Introduction

Influenza A H3N8 viruses have been originally isolated from birds, nowadays these viruses are currently the main causal agent of respiratory diseases in horses [1, 2] and their transmission from equines to dogs has already been observed [2, 3]. These enveloped viruses belong to the *Alphainfluenzavirus* genus into the *Orthomyxoviridae* family [4], which present a segmented genome of single-stranded negative RNA.

Their envelope exposes haemagglutinin (HA) and neuraminidase (NA) glycoproteins, rod-shaped trimeric and mushroom-shaped tetrameric spikes, respectively [5]. The haemagglutinins are classified into 18 subtypes and composed of head, where the receptor binding and the antigenic sites are localized, and a long fibrous stem, where a cleavage site is localized that, by its amino acid composition, permits classification of the virus samples by their pathogenicity in low pathogenicity avian influenza (LPAI) and highly pathogenic avian influenza (HPAI). The neuraminidase glycoproteins are classified into 11 subtypes and composed of head, where the sialidase cleavage site on sialic acid residues and the antigenic sites are localized, and a thin long stalk [5–7].

These viruses have been isolated from humans and a variety of animals, including, pigs, horses, sea mammals, poultry, wild ducks, birds and bats [7, 8], causing respiratory infections that result in severe human and animal suffering and high economic losses [9]. Only the genus *Alphainfluenzavirus* of influenza viruses are known to cause natural infections in birds; and the viruses are classified by their ability to cause avian disease as LPAI and HPAI samples. The latter is involved in outbreaks in birds and humans worldwide [10].

Vaccines are a recommended strategy for controlling these infections in humans and animals. Hydrostatic pressure (HP) has been a valuable tool to disturb the structure of many viruses and to allow a better understanding of the physical and chemical processes taking place in these particles [11, 12]. HP as a nonthermal, additive-free preservation method can be used to inactivate viruses [13]. The pressure-induced dissociation of viral proteins and icosahedral capsids is a phenomenon that has been demonstrated in many viruses [14–16]. The effects of the hydrostatic pressure have been studied [14] and used to inactivate some non-enveloped viruses presenting icosahedral capsid, such as hepatitis A virus (HAV) [17], foot-and-mouth disease virus (FMDV) [18, 19], norovirus [20, 21], infectious bursal disease virus (IBVD) [22] and rotavirus [23, 24]. This inhibitory effect of HP have been also shown on enveloped viruses of icosahedral capsid such as simian immunodeficiency virus (SIV) [25, 26], some strains of human immunodeficiency virus (HIV) [15, 25], and Mayaro virus [27], classified into the *Alphavirus* genus such as the Chikungunya virus. This effect has also been demonstrated on enveloped viruses of helicoidal capsid such as vesicular stomatitis virus (VSV) [24, 28].

Promising results have already been obtained with pressurized and inactivated influenza A H3N8 virus in studies that support the idea that non-human viruses can be used to produce vaccines for humans [29–31]. Here, we describe the effects of hydrostatic pressure (from 0 to 2.9 kbar) on the structure, morphology and functional characteristics of avian influenza A H3N8 virus by using fluorescence spectroscopy, light scattering and electron microscopy. For functional analyses, the viruses were assayed for biological activities of their haemagglutinin and neuraminidase spikes. Our study provides a detailed description of the relationship between the structure stability of avian influenza virus and its inactivation by HP treatment.

## Methods

### Chemicals

Urea and guanidine were purchased from Merck KGaA (Darmstadt), while bis-8-anilino-1-naphthalenesulfonate (bis-ANS) was acquired from Thermo Fisher Scientific (Waltham, MA, USA). All other reagents were of the highest commercially available analytical grade. Distilled water was filtered and deionized through a Millipore water purification system.

### Influenza virus preparation

Avian influenza A/duck/Ukraine/1/63 (H3N8) sample was replicated for 24 h at 37 °C in the allantoic cavity of 10-day-old embryonated chicken eggs. The allantoic fluid was collected, and cell debris was removed by a low-speed spin (6000 *g*). The virus was pelleted by spinning the allantoic fluid at 80 000 *g*, then resuspended in TE buffer (20 mM Tris, 2 mM EDTA, pH 8.4), and banded at 100 000 *g* on a continuous 20–60% sucrose density gradient in TE buffer, pH 8.4. The protein concentration of the purified virus sample was determined as preconized by Lowry *et al*. [32] and stored at −80 °C after confirmation of its purity by electrophoresis on 12.5 % SDS-polyacrylamide gel [33].

### Structural analysis by fluorescence and light scattered

The HP cell has been described by [34] and was purchased from ISS (Champaign, IL, USA). It was coupled to a fluorimeter and equipped with sapphire windows [35]. Fluorescence and light-scattering measurements were recorded on ISSK2 spectrofluorometers (ISS. USA). Samples were excited at 280 nm and emission was observed from 300 to 420 nm. Scattered light (320 nm) was collected at an angle of 90° of the incident light by integrating the intensity in the 315–325 window. In order to detect exposure of hydrophobic segments of influenza virus proteins, the fluorescent probe bis-ANS (15 µM) was excited at 360 nm and the emission of its extrinsic fluorescence was measured between 400 and 600 nm.

### Structural analysis by electron microscopy

Virus preparations were observed in a Morgani electron microscope, 100 KV operating voltage. Negative staining was performed with 2.0 % uranyl acetate as described. The samples were placed on copper grids coated with carbon film, the concentration of 400 µg ml^−1^.

### Functional analysis of hemagglutinating and sialidase activity

For analysis of their haemagglutinating (receptor-binding) activity, virus preparations were assayed by haemagglutination assay in 96-well micro-titre plates (Nunc, U type), 25 µl of PBS was added to each well. Viral suspension (25 µl) was added to the first well in column one, serial dilutions were made by transferring 25 µl from the first well of column one to the successive columns, the final 25 µl were discarded. The positive control was done with lectin and negative with PBS. Lastly, 25 µl of 0.5 % human erythrocyte suspension was added to each well on the plate and haemagglutinating litres were recorded after 45 min as previously described [36]. For analysis of their sialidase activity, virus preparations were assayed in 96-well micro-titre plates (Corning, black flat botton type) with 4-methylumbelliferyl-Nacetyl-a-d-neuraminic acid (4-MU-NANA) ammonium salt (Nacalai Tesque, Kyoto, Japan), according to a fluorometric assay method as described previously [37]. Five microlitres of viral suspension, 20 µl of a solution of NA inhibitor, and 20 µl of 0.1 mM 4-MU-NANA solution were mixed and incubated for 60 min for 37 °C. Fluorescence of released 4-MU was measured with excitation at 365 nm and emission spectra were recorded at 450 nm from fluorescence spectrophotometer SpectraMax M5 (Molecular Devices).

### Virus infectivity assay

The infectivity of the influenza virus was tested with 50 % tissue culture infectious dose (TCID_50_) in Madin-Darby Canine Kidney (MDCK) cells obtained from the Rio de Janeiro Cell Bank. Confluent monolayers of MDCK cells were infected with serial dilutions of 10^−1^ to 10^−7^. After 48 h at 37 °C, the cytopathic effects of influenza virus were observed under microscope and TCID_50_ was calculated according to the Reed and Muench method [38].

### Blind infection to access residual virus infectivity

The residual infectivity of the pressurized virus samples was assayed for three sequential blind passages. For each blind passage, the samples, which had no infectivity by TCID_50_, were inoculated into eggs (five eggs per sample) and incubated, as described previously. Finally, the collected and clarified allantoic fluids were analysed by hemagglutination test [HI].

## Results

**Fig. 1.**
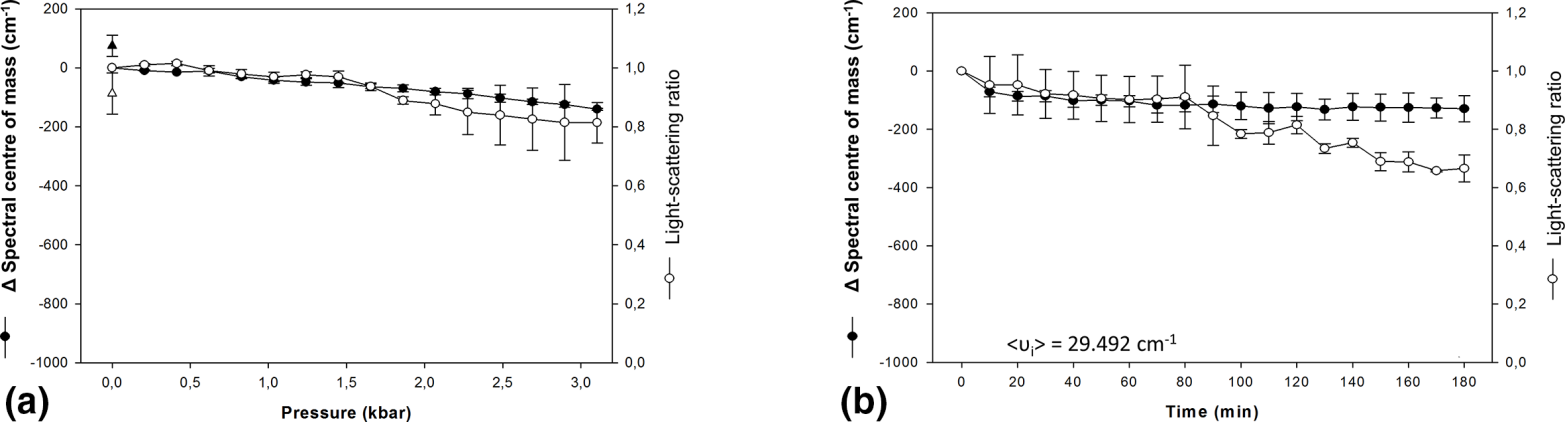
Effect of high hydrostatic pressure treatment on conformation of influenza A H3N8 viruses. The shift in the centre of spectral mass (●) and changes in light scattering (○) were evaluated throughout gradual increases of pressure (a) and as a function of 2.9 kbar applied for 3 h (b). Fluorescence data points in both graphs include the average and standard deviation results of three experiments and light-scattering curves are representative of three measurements. Points represent the mean of three independent experiments and the bars the standard error.

Previous studies have shown that hydrostatic pressure can be used to inactivate enveloped and non-enveloped RNA and DNA viruses [39–42]. In this study, we show that avian influenza H3N8 particles submitted to this treatment undergo changes in conformation, morphology and size. The effects of pressure on the tertiary conformation of the influenza viruses were analysed by changes on the intrinsic fluorescence spectra and light scattering of the particles. Fig. 1a shows the effects on the intrinsic fluorescence and light scattering of the influenza A H3N8 virus particles by applying gradual pressure. By applying higher pressure, the variation of the centre of spectral mass decreased, resulting in a total spectral red shift of about 100 cm^−1^. This result indicates that few conformational changes have occurred to the exposed particles. Our data reveal a decrease of approximately 20 % in LS when pressure reached around 3 kbar.

**Fig. 2.**
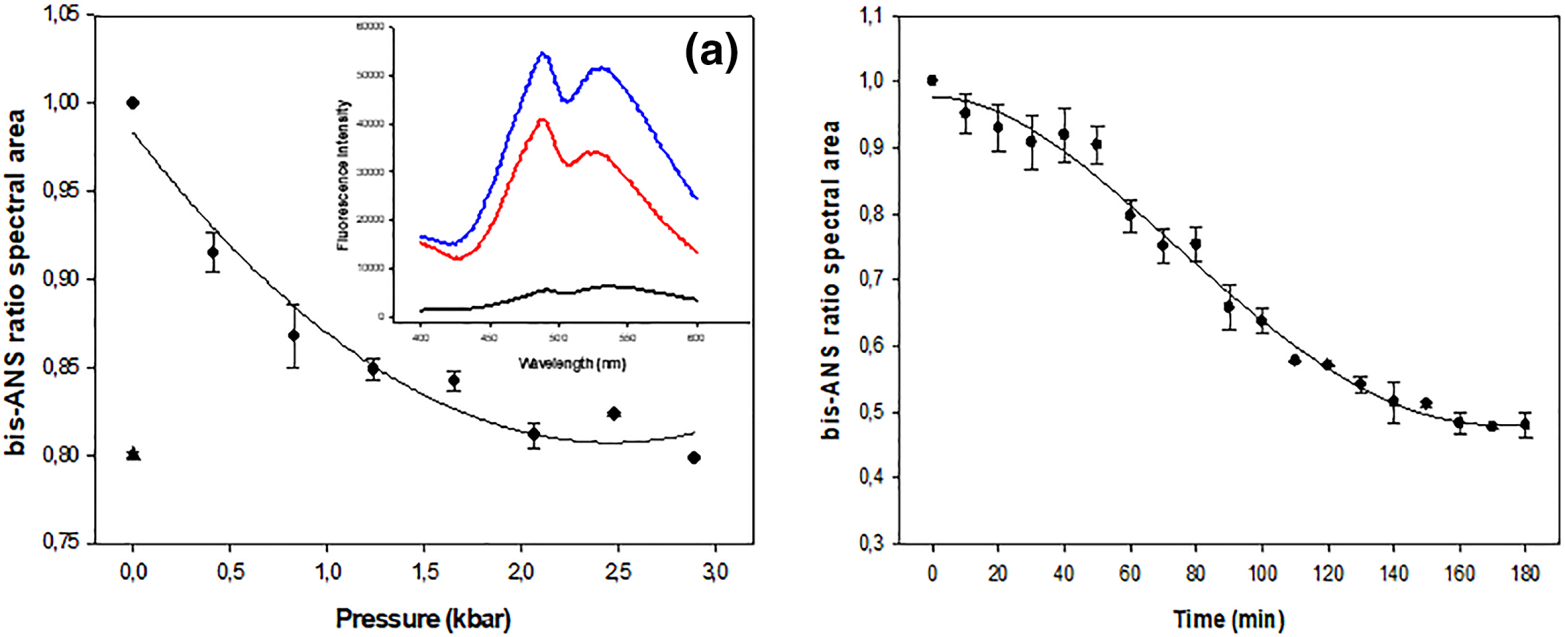
Effect of high hydrostatic pressure treatment on bis-ANS binding to influenza A H3N8 viruses. The viruses were pre-incubated for 10 min with 15 mM bis-ANS probe. (a) The virus was submitted to a gradual pressure increase until it reached 2.9 kbar. The incubation time at each pressure point was 10 min. (b) The viruses were submitted to 3.0 kbar and the probe intensity checked every 10 min until it reached 180 min. Points represent the mean of three independent experiments and the bars the standard error. Inset: fluorescence emission spectra of influenza viruses: Under pressure (red line) and native particles (blue line). Black line is bis-ANS probe free in solution.

**Fig. 3.**
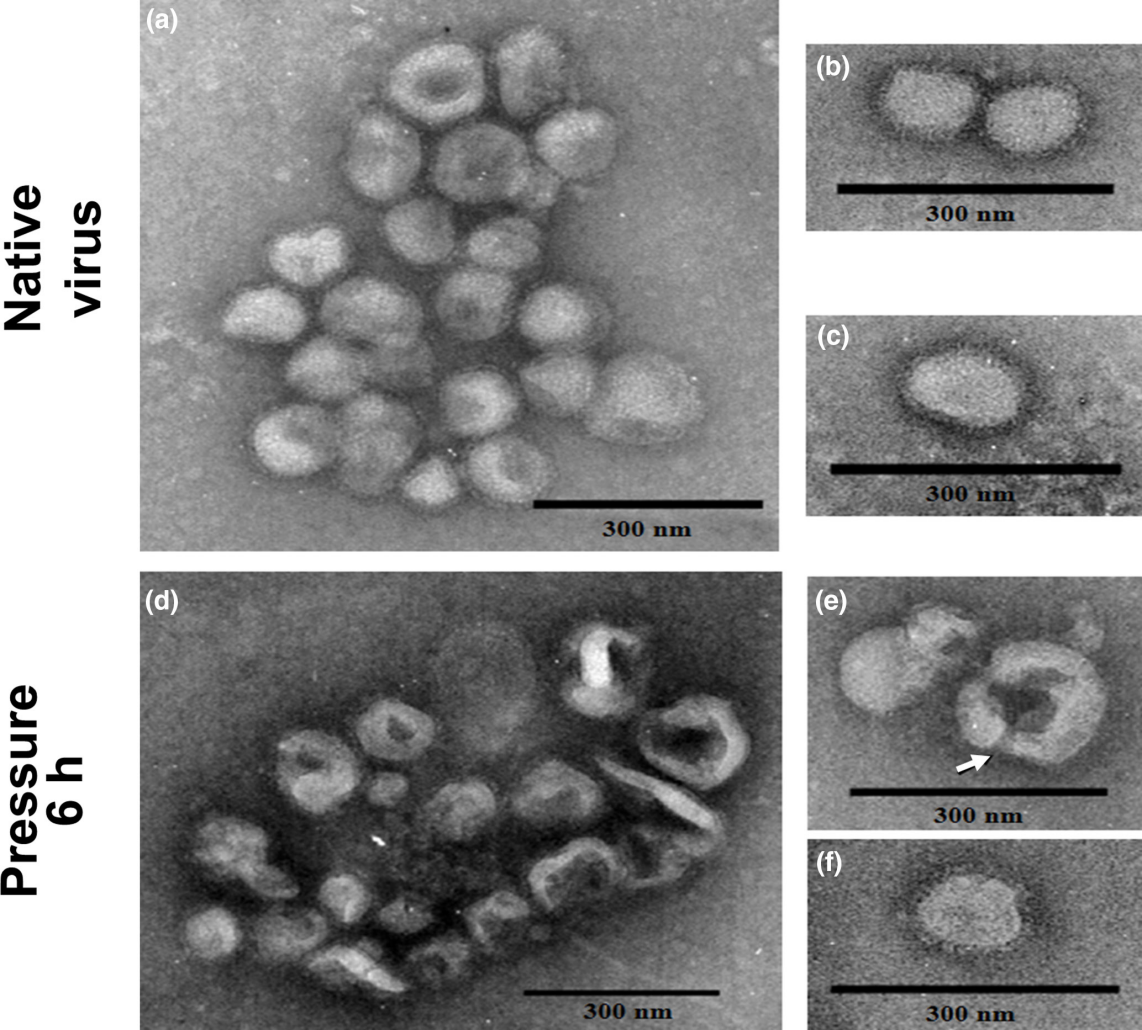
Effect of high hydrostatic pressure treatment on the morphology of influenza A H3N8 viruses. Purified preparation of viruses (400 µg ml^−1^) pressurized for 6 h/2.9 kbar at 25 °C was morphologically analysed by electron microscopy. (a–c) Control samples and (d–f) pressurized samples. The arrow indicates the formation of a ‘pore’ on the viral envelope. The selected micrographs are representative of all grids analysed. Bars: 300 nm.

The effects of this pressure range applied for 6 h resulted in a variation in the centre of spectral mass of about 300 cm^−1^ (Fig. 1b). This single elevated pressure also affected the light-scattering values of influenza viruses. Fig. 2a shows a 20 % decrease of bis-ANS fluorescence when gradual increasing pressure is applied, leading to a slight detachment of the probe. When the pressure was raised to 3 kbar and kept at this pressure for 6 h, bis-ANS fluorescence yield gradually decreased. To approach the morphological changes, we access the transmission electron micrographs of influenza virus after incubation at 2.9 kbar (289.58 Mpa – 42 kpsi –2895.8 bar) (25 °C for 6 h) as compared to controls incubated at atmospheric pressure. The native influenza virus is a pleomorphic particle, as shown in Fig. 3(a–c). However, viruses treated by high pressure had the same size as their native counterparts, but their shells were not as continuous or regular as the latter (Fig. 3d).

Many pressurized virus particles showed discontinuing envelopes (Fig. 3d), other presented spikes in their envelopes (HA and NA) and had their architecture visibly altered (Fig. 3f). Additionally, virus particles were not fused. However, it is interesting to note that many of these particles presented pores in the envelope and showed leakage of internal content or complete absence. However, some whole virus particles were observed after this pressurization process (Fig. 3e). These electron micrographs suggest that HHP affects influenza virus structure.

**Fig. 4.**
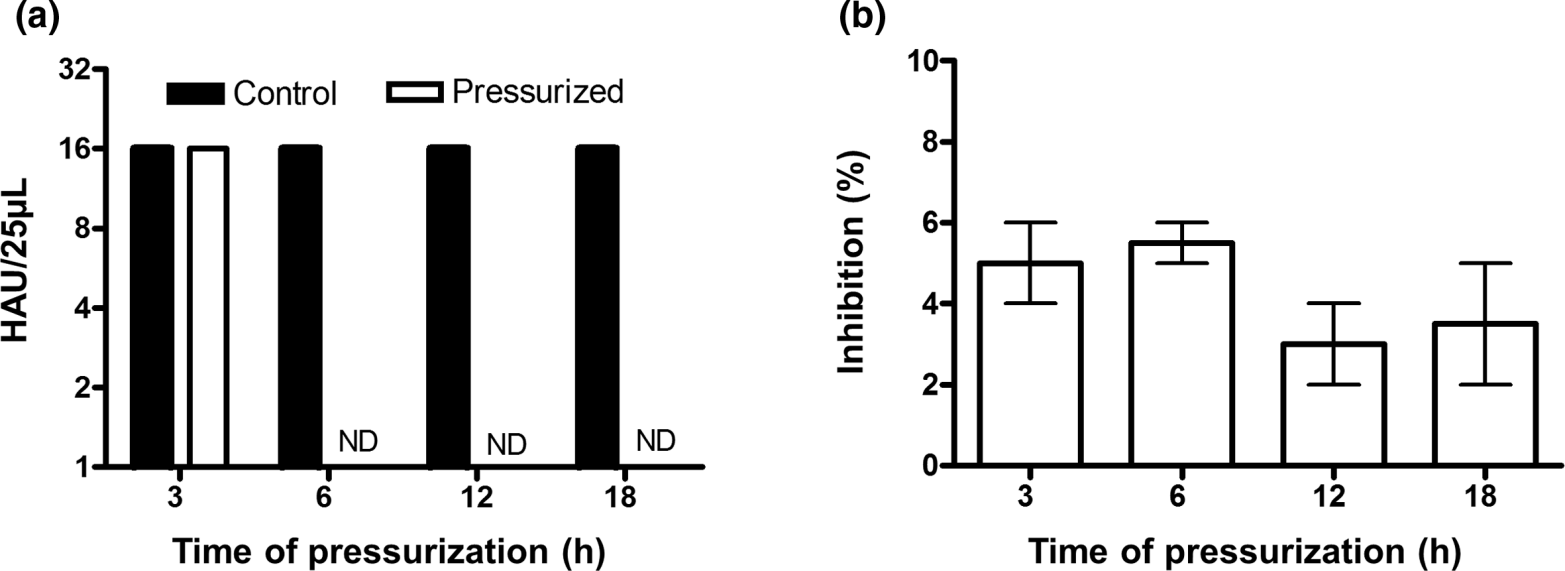
Effect of high hydrostatic pressure treatment on HA and NA biological activities of influenza A H3N8 viruses. (a) Haemagglutinating litres of pressurized viruses at pH 7.4 for 3, 6, 12, 18 h at 3.1 kbar. Haemagglutination units (HAU) were determined by the reciprocal of the highest dilution where total haemagglutination was observed. (b) Inhibition of neuraminidase activity of pressurized virus at pH 7.4 for 3, 6, 12, 18 h at 3.1 kbar was determined by fluorimetric assay using MU-NANA as substrate. The NA activity of the pressurized virus was calculated by normalizing NA activity to the level of the native virus. ND, Not detected.

**Fig. 5.**
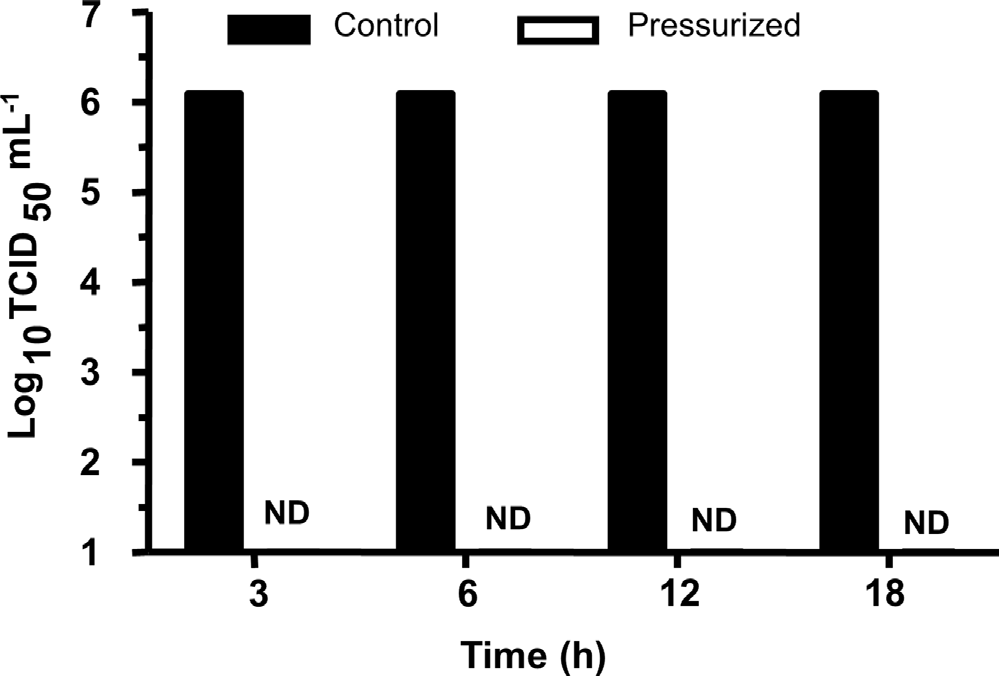
Effect of high hydrostatic pressure treatment on the infectivity of influenza A H3N8 viruses. In this assay, purified influenza virus preparation at a concentration of 100 µg ml^−1^ was pressurized during 3, 6, 12 and 18 h at 2.9 kbar at 25 °C and titred for its infectivity (TCID_50_) in MDCK cells. ND, not detected by the method used.

To investigate the effect of high pressure in the haemagglutinin activity we applied the haemagglutination test. Our data show that 3 h of applied pressure did not reduce the haemagglutinating activity of the virus particles. However, additional time of pressurization led to a complete loss of the haemagglutinating titre (Fig. 4a), suggesting that pressure leads to some structural changes that abolish the haemagglutinating activity of the particles. We also investigated the NA activity that showed no significant inhibition of its sialidase activity when under pressure at any of the four times here tested (3, 6, 12 and 18 h) (Fig. 4b). Several studies describe the effect of pressure in reducing infectivity and in turning the virus inactive both in enveloped and non-enveloped viruses with minimal effects on the integrity of the particles. Fig. 5 shows the pressure effect (at 25 °C) on the infectivity of influenza H3N8 viruses that was determined by TCID_50_ on MDCK cells. As shown in this figure, applied pressure for all of the four times tested here (3, 6, 12 and 18 h) dramatically decreased the virus litres in all cases.

**Fig. 6.**
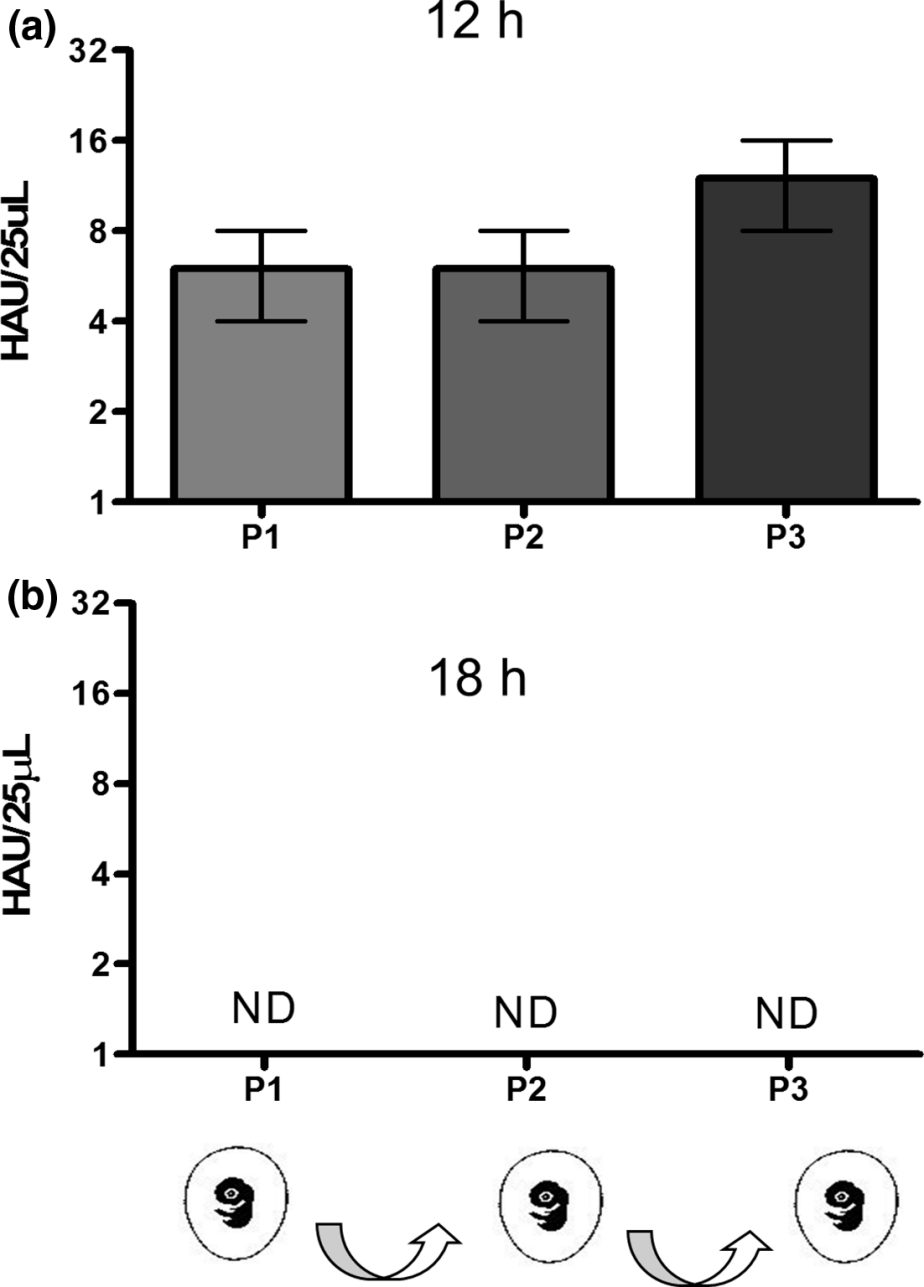
Analysis of potential residual infectivity of influenza A H3N8 viruses. Influenza virus preparation submitted at pressure-induced inactivation process was analysed by three serial passages (P1, P2 and P3) in embryonated chicken eggs. The samples were pressurized for 12 h/2.9 kbar (a) and 18 h/2.9kbar (b) at 25 °C and then analysed by haemagglutination assay. UHA, hemagglutinating units; ND, not detected.

Entire virus particles that underwent pressurization were tested for a potential residual infectivity. For that, the pressurized preparation of virus particles was submitted to three serial passages in embryonated chicken eggs. Assay titration of pressurized viruses for 6 h showed an increase in titre after the first passage, which became higher at second and third passages (Fig. 6a). These results indicate that the intact particles observed in this pressurized sample were able to replicate and thus had a residual infectivity. We next increased the pressurized time for 18 h, and the replicate ability of these particles was then lost, as revealed through the haemagglutination assay done after each of the three serial passages (Fig. 6b).

## Discussion

Our results show that the influenza virus has undergone conformational changes promoted by pressure as well as changes in function of their membrane glycoproteins. These observations are consistent with data showing that high hydrostatic pressure is able to promote modifications in the viral structure, which can lead to virus inactivation.

Non-enveloped RNA viruses such human rhinovirus type 14 (HRV14) [34], foot-and-mouth disease virus (FMDV) [18, 19], infectious bursal disease virus (IBVD) [22] and rotavirus, and enveloped RNA viruses such as vesicular stomatitis virus (VSV) [24, 28], avian metapneumovirus [24] and influenzavirus, subject of our analysis, are examples of infectious agents inactivated by pressure.

The effect of applying hydrostatic pressure for 3 h on H3N8 virus revealed that the particles were resistant to this condition, and the virus displayed only a discrete conformational change, as observed by bis-ANS fluorescence (Fig. 2). Additionally, we observed a small decrease in the value of the centre of mass, which indicate a small shift in fluorescence emission spectrum of tryptophan (Fig. 1a). Lastly, no significant changes in values of light scattering were observed.

The pressure of 3 kbar alters the exposure of tryptophan residues of viral particles. Under the same conditions, the human rhinovirus 14 (HRV14) showed deviations of 350 cm^−1^ [43], the FMDV varied 200 cm^−1^ [19], and IBDV varied 690 cm^−1^ [22]. These differences in variation suggest that viruses have a certain ‘individual thermodynamics’ and this behaviour seems to be the result of interactions among viral proteins and between those and the viral genetic material [44].

This discrete structural change caused by hydrostatic pressure leads to virus inactivation. In the case of other pressure-inactivated viruses, such as vesicular stomatitis virus (VSV) or rotavirus, constant changes on the shape of viral particles and on the integrity of the membrane or the coat proteins were also observed [35, 45]. Enveloped virus particles have a high level of complexity, due to the multiple interactions of the capsid with the nucleic acid and with the associated lipids in the viral membrane. To characterize the morphology of the particles, electron microscopy experiments were performed with the pressurized samples for 6 h and pores were observed crossing the envelope of these treated samples (Fig. 3). However, a small number of whole particles could be observed by electron microscopy (Fig. 3f). The presence of pores and the observed alteration in the display of glycoproteins in the virus envelope might be explained by the fact that hydrostatic pressure is known to promote a decrease in lipidic fluidity of biological membranes [46], (Fig. 3e).

During an influenza virus infection, the adsorption promoted by haemagglutinin glycoprotein is a function of its binding to sialic acid receptor sites [47]. Our data show that a pressurization time longer than 3 h led to complete loss of the haemagglutination titre (Fig. 4a), suggesting that pressure leads to some structural changes in the haemagglutinin, resulting in loss of its cell-binding activity. The neuraminidase was evaluated *in vitro* for its sialidase activity, which allows the removal of sialic acid residues from both haemagglutinin and neuraminidase spikes, as well as from cell surface glycoconjugates. Interesting, the pressure range used was not able to cause significative effect on the neuraminidase activity in all periods of time tested (Fig. 4b). Palese *et al.* [48] have shown that an impaired function of neuraminidase activity may result in aggregation of virus particles appearing on the surface of the host cell. This observation corroborates the electron microscopy images in which no aggregation in pressurized virus is noticed (Fig. 3d). The resistance of the NA mushroom-shaped structure to the hydrostatic treatment can be explained by its cluster arrangement on the virus envelope and by the fact that its globular dense heads are less exposed.

We found that pressure of 3 kbar applied for 6 h was able to inhibit haemagglutination and infectivity while virus replication was no longer observed, suggesting that full virus inactivation occurred at this point. However, this inhibition was observed in the presence of NA activity that was only slightly affected at 18 h (Fig. 4b), suggesting that inhibition of NA sialidase activity is not a *sine qua non* condition for virus inactivation. This unexpected finding deserves further investigation.

On the other hand, the fact that haemagglutination was impaired by pressurization agrees with other results previously reported for other subtypes of influenza A viruses (H3N2) [49] and H3N8 [50] suggests that the virus-binding activity promoted by HA is primordial for the virus infectious process. The long period of pressurization required for full inactivation of the influenza viruses agrees with findings reported by Silva *et al.* [39], revealing that this procedure was also crucial to achieve the full inactivation of another enveloped virus as VSV. Enveloped viruses with helical structure are highly destabilized by high pressure, that leads their glycoproteins to assume a receptor-activated conformational state, mimicking a fusion-active conformation [40]. Perhaps, as already observed to VSV, this fusion-active conformation prevents the internalization by endocytosis [40].

The structures of the VSV were not dissociated after a long time of pressurization, but the subunits appear displaced from their normal positions, as shown by bulges under the envelope [38]. Pressure was also able to inhibit the haemagglutinating activity of dimeric VP4 spikes (receptor-binding structures) of rotavirus, a triple-shelled non-enveloped virus, that loses its infectivity but maintains its immunogenicity [23].

Studies with pressure-inactivated particles of yellow fever virus [44], infectious bursal disease virus [22], vesicular stomatitis virus (VSV) [35], simian and bovine rotavirus [23] and human influenza virus [49, 50] demonstrate that they maintain their immunogenic properties as native particles. These interesting findings have generated the idea of applying hydrostatic pressure to prepare virus vaccines with high efficiency and safety. In addition, it is also a simple and inexpensive means to prepare effective vaccines [22, 35, 44, 51]. As a physical approach, high pressure shows many advantages over other methods of preparing vaccines. The application of pressure does not introduce exogenous substances into vaccines as chemical methods usually do.

The findings of this study showing that pressure does not act equally on HA and NA glycoproteins reveal that the technique presents some selectivity that might be a useful feature when considering it for developing a potential inactivated viral particle, as some desirable parts of the virus may still remain functional. For instance, whole enveloped particles of pressurized virus may preserve, probably by very subtle structural changes [38], the immunogenicity of their original antigenic structure. Recent exciting findings, that need to be discussed, show that antibodies to HA can function *in vivo* by blocking NA enzyme activity to prevent virion release and enhance Fc receptor-based activation of innate immune cells [52]. Our data bring more information to the field of structural virology of enveloped particles and reinforce the idea of applying hydrostatic pressure to prepare antiviral vaccines.
